# Authors’ Reply to McMahon et al. Comment on: “Anthropometric and Physical Qualities of Elite Male Youth Rugby League Players”

**DOI:** 10.1007/s40279-017-0770-7

**Published:** 2017-08-17

**Authors:** Kevin Till, Sean Scantlebury, Ben Jones

**Affiliations:** Leeds Beckett University, City Campus, Leeds, LS1 3HE UK

Dear Editor,

We would like to thank McMahon et al. [1] for their kind and constructive comments on our recent review article [2]. After reading the comments by McMahon et al. [1], we fully acknowledge that we did not discuss the validity of the methods used to derive countermovement jump (CMJ) height within our review. Therefore, the comments by McMahon et al. [1] do supplement the review article with important information for research and practice within rugby league and other sports.

The valid comments provided by McMahon et al. [1] relating to the CMJ height are not the only methodological considerations that should be applied to our review paper [2]. For example, aerobic capacity is an estimate of maximum oxygen uptake (i.e. estimated VO_2max_) predicted from field-based tests [e.g. multistage fitness test (MSFT) and Yo-Yo intermittent recovery test level 1 (Yo-Yo)]. Prediction equations derived from these field-based tests to estimate VO_2max_ inherently induce a degree of error [3]. Furthermore, due to advances in knowledge and methodologies within research, it is also important to note the changes in testing batteries used, and how data are reported. For example, recently Yo-Yo test data have been reported as distance covered [4, 5], as opposed to estimated VO_2max_ in earlier studies [6]. This is represented in Table 2 in Till et al. [2], which presents four types of aerobic capacity data (i.e. MSFT estimated VO_2max_, MSFT level, Yo-Yo estimated VO_2max_ and Yo-Yo distance) based on recent changes within the literature. These methodological considerations highlight the importance of critically evaluating the current literature by researchers and practitioners alike.

Regarding the CMJ specifically, it now appears more common for researchers in the field to use a force platform for the collection of CMJ height (e.g. Darrall-Jones et al. [5], Ireton et al. [7] and Roe et al. [8]) as opposed to the methods (i.e. Takei Vertical Jump Meter, Just Jump System, and Yardstick Jump Testing System) highlighted by McMahon et al. [1]. This suggests that within the assessment of physical qualities, researchers are applying scientifically valid measurements where possible (e.g. equipment availability) to increase understanding of performance, which is likely due to the increased availability of scientific equipment. Regardless of the equipment used within studies, we recommend that when both researchers and practitioners apply data from the literature to their cohort, they should establish their own population-specific typical error and smallest worthwhile change reliability data to determine meaningful changes [9].
